# Forged by charge: polaron-induced matrix formation in silicon nitride conversion-type anodes for lithium-ion batteries

**DOI:** 10.1039/d5ta04013b

**Published:** 2025-08-04

**Authors:** Jonathon Cottom, Lukas Hückmann, Jörg Meyer, Emilia Olsson

**Affiliations:** a Institute for Theoretical Physics, University of Amsterdam Science Park 904 Amsterdam 1098 XH The Netherlands k.i.e.olsson@uva.nl; b Advanced Research Center for Nanolithography Science Park 106 1098 XG Amsterdam The Netherlands; c Leiden Institute of Chemistry, Gorlaeus Laboratories, Leiden University P. O. Box 9502 2300 RA Leiden The Netherlands

## Abstract

The quest for high-capacity anode materials is vital in developing future lithium-ion battery technologies. While silicon-based anodes offer high theoretical capacity, their commercial realization is hindered by instability associated with large volume changes. Amorphous silicon nitride (a-Si_3_N_4_) has emerged as a promising alternative, acting as a conversion-type anode where lithium incorporation drives the formation of a structurally robust matrix and active phases. Here, we demonstrate that charge trapping, driven by polaron and bipolaron formation, governs the structural transformation of a-Si_3_N_4_ during the initial lithiation. These charge-induced modifications lead to the formation of a Li–Si–N matrix that stabilizes the anode framework. Matrix generation is accompanied by the development of Si-rich regions, serving as precursors for the active phase. We identify a progression from electronically active polarons to inactive bipolaron states, establishing a direct link between charge localization and matrix formation. These insights recast charge trapping from a passive consequence to a functional design parameter for optimizing conversion-type anodes.

## Abstract 2

Charge-trapping in silicon nitride anodes govern structural transformations during the initial lithiation.

## Graphical abstract

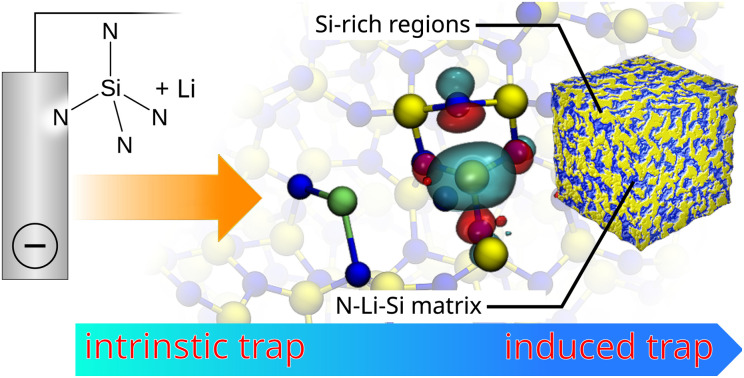

## 1 Introduction

The development of more efficient battery technologies is a critical enabler of the green transition and the shift towards a circular economy. Metal-ion batteries occupy a central role in this transformation, underpinning energy storage and delivery across a wide range of applications – from renewable energy integration and grid-level storage to electric vehicles and personal electronic devices.^1–3^ Looking ahead, two key challenges must be addressed by next-generation metal-ion batteries: increased anode capacity and improved cell lifetime.^4–10^ These interdependent performance metrics remain central obstacles in the search for advanced anode materials.

These challenges have driven the search for new anode materials with both higher capacity and improved cycle life.^5,10–13^ Since the early development of lithium-ion batteries, graphite has dominated as the anode material of choice due to its stability, despite a limited capacity of only 360 mA h g^−1^.^4–9^ Silicon, an alloying-type anode, has long been considered the natural successor to graphite, offering a theoretical capacity of 3579 mA h g^−1^.^13–18^ However, Si anodes suffer from severe structural degradation caused by a volumetric expansion and contraction of approximately 300% during lithiation and delithiation.^18–21^ These extreme volume changes lead to mechanical failure of the composite cell, as other components are unable to accommodate the deformation.^13,22^ In response, various structuring strategies have been explored to mitigate mechanical stress in silicon-based systems.^16,17,22^ In parallel, attention has shifted towards alternative materials that form a Si-rich active phase *in situ*, particularly sub-stoichiometric oxides and more recently nitrides.^17,23^ In these systems, the first lithiation step induces an irreversible conversion that yields both an electrochemically active Si-rich phase and an inert embedding matrix.^17,20^ This composite structure has been shown to improve mechanical resilience and provide stable cycling performance after the initial transformation.^24^ However, the mechanism underlying this initial conversion remains poorly understood.

Silicon nitride has been a technologically important material long before its consideration as a next-generation anode candidate, being employed in a broad range of applications including wear-resistant coatings,^25^ electronic devices (ReRAM, MOSFETs, and MEMS),^26–28^ high-energy optics,^29–31^ and integrated photonics.^32,33^ Almost universally, Si_3_N_4_ is deployed as an amorphous thin film (a-Si_3_N_4_).^27,28,34–38^ In battery applications, silicon nitride has been studied across a range of structures and stoichiometries, and prepared using a variety of growth methods.^19,23,39–48^ These diverse approaches have yielded a wide range of reported lithiation capacities, from 40 mA h g^−1^ to 2000 mA h g^−1^. In general, the highest capacities are observed in silicon-rich SiN_*x*_ compositions, although this comes at the expense of cycling stability. The optimal reported stoichiometry is approximately SiN_0.9_, which delivers a capacity of 1200 mA h g^−1^ with stable performance over more than 2000 cycles.^45,46^ As stoichiometric a-Si_3_N_4_ is approached, capacity declines sharply. Conversely, further increases in silicon excess result in rapid capacity fade over just tens to hundreds of cycles.^45^ Many explanations have been proposed for this tradeoff, but a definitive link between macroscopic performance and atomic-scale Li incorporation mechanisms remains an open question.

Recent studies of silicon nitride anodes have revealed key insights into the formation and stability of the matrix that emerges during the first lithiation cycle. Early reports proposed the formation of a Li_3_N phase, motivated by thermodynamic considerations and observed improvements in ionic conductivity,^19^ though no direct structural characterization was available. More recent work by Ulvestad *et al.*,^47^ employing Pair Distribution Function (PDF) and Energy Dispersive X-ray (EDX) analyses, suggests that the matrix is better described as a mixed Li–Si–N network, with the best-fit composition approximated by Li_2_SiN_2_ stoichiometry. Concurrent EDX mapping revealed a compositional segregation of the original SiN_*x*_ network into Si-rich domains and nitrogen-enriched amorphous lithium–silicon–nitride regions. In contrast, Kilian *et al.*^43^ employed ^7^Li NMR to challenge the picture of a static inert matrix, instead suggesting that silicon nitride forms a redox-active Li–Si–N solid solution with evolving local environments throughout cycling. Lovett *et al.*^49^ further confirmed the domain behavior and demonstrated both the mechanical robustness of the matrix and its stabilizing influence on the active (Si-rich) phase. As exemplified by this ongoing debate, definitive identification remains challenging due to the amorphous character of the matrix, local compositional variations, and potential electrochemical activity. These factors underscore the importance of elucidating the atomic-scale mechanisms that govern matrix formation and evolution.

Despite these important advances, the underlying mechanisms driving matrix formation and stability remain poorly understood. In particular, the atomic-scale processes that govern the initial lithiation of silicon nitride, and the influence of the local atomic environment on lithium incorporation and storage, are not yet resolved. The evolution of the matrix as a function of lithium concentration, and its dependence on the host network structure, are especially difficult to probe *via* post mortem measurements, since key features of the initial lithiation state are often obscured by subsequent structural relaxation. Furthermore, the relationship between the microstructure of the silicon nitride precursor – including its nanoporosity and local topology – and the morphology of the emerging matrix warrants deeper investigation. Addressing these questions is essential not only for optimizing silicon nitride-based anodes, but also for advancing the broader understanding of conversion type anodes for next-generation lithium-ion battery materials.

In this work, we present a systematic study of lithium incorporation in a-Si_3_N_4_, focusing on the initial steps of this process at the atomic scale. To ensure that a statistically meaningful range of incorporation sites are considered, we build on the sampling scheme developed in our previous work.^50,51^ Using density functional theory (DFT) simulations, the stability, geometry, and interplay between the Li ions and intrinsic charge trapping^50^ is probed. Our study provides a detailed atomic-scale understanding of the irreversible matrix formation and elucidates concomitant reversible charge trapping.

## 2 Computational methods

All DFT calculations were performed spin-polarized using the CP2K^52^ code with the HSE06 (ref. 53 and 54) exchange-correlation functional in combination with the auxiliary density matrix method (ADMM),^55^ which reduces the computational cost of hybrid functional calculations. The DZVP-SR-MOLOPT^56^ family of basis sets was employed to describe the valence electrons, together with GTH pseudopotentials^57–59^ for the core electrons. Energy cutoffs were set after convergence testing to 650 Ry and 70 Ry for the relative cutoff, yielding a precision of 0.1 meV per atom. All geometry optimisations were performed using the BFGS algorithm,^60–63^ with convergence criteria of 10^−7^ eV for energy differences and 0.001 eV Å^−1^ for forces.

Using the standard formalism of Zhang and Northrup^64^ at the hybrid functional level, average defect formation energies per lithium atom incorporated into a-Si_3_N_4_ were calculated according to1
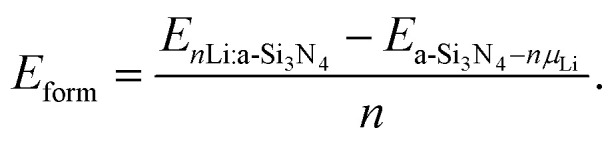
Here, *E*_a-Si_3_N_4__ and *E*_*n*Li:a-Si_3_N_4__ are the DFT total energies of the a-Si_3_N_4_ simulation cell without and with *n* inserted Li atoms. The chemical potential of Li (*μ*_Li_) is taken from bulk lithium in its ground state body-centred cubic phase. Other common references that can be found in the literature are EC/DMC solvated Li^+^ ions or Li^0^ atoms in vacuum. Due to reduced Li–Li interaction the corresponding values for *μ*_Li_ are ≈1.0 eV and ≈1.8 eV lower, respectively.^65^*E*_form_ as defined in eqn (1) can also be read as average incorporation energy for the *n* inserted Li atoms relative to bulk Li. An alternative approach would be to calculate the formation energy in a “step-wise” manner where the formation energy for Li_*n*_ is referenced to Li_*n*−1_. While this has no impact on the reported trends, the energy range is expanded as there is no averaging over Li sites in this approach (as in each case *n* = 1), this treatment is shown in Fig. S6 in the SI.

**Fig. 1:**
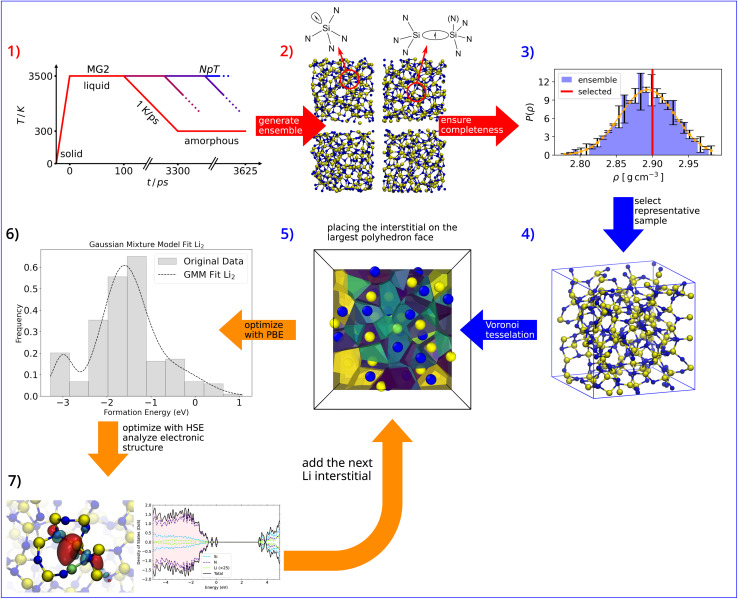
Computational workflow with the key stages highlighted: 1 and 2 are the structure generation *via* an MD melt quench,^50^ highlighted in red. Stage 3 represents the statistical convergence of the ensemble with respect to a given descriptor space, and stage 4 and 5 (highlighted with blue numbers) represent the site sampling *via* Voronoi tesselation.^51^ Stage 6 and 7 (in black) iteratively repeat an exhaustive PBE screening followed by a GMM sampling and finally the HSE06 production run for a given Li concentration.

The primary focus of this work is on Li incorporation in amorphous silicon nitride (a-Si_3_N_4_), where the vast configuration space necessitates the multi-stage sampling workflow shown in Fig. 1. To contextualize the results for the range of sites found in the amorphous system, calculations for Li_1_ were performed on crystalline β-Si_3_N_4_ to serve as an ordered reference model. The following sections detail the generation and sampling of the amorphous structures, while a full description of the crystalline calculations can be found in the SI (see S1 and S2).

The amorphous host network is first generated *via* a classical melt-quench molecular dynamics trajectory. A 280-atom β-Si_3_N_4_ supercell was melted and then cooled at a rate of 1 K ps^−1^ under an isothermal–isobaric (NpT) ensemble using the MG2 interaction potential,^66^ following the protocol in ref. 50 (Fig. 1, stages 1–2 highlighted in red). As structure sampling represents a vital consideration for amorphous systems, an ensemble is generated that is statistically converged with respect to a defined descriptor space. From this ensemble a single, statistically representative 280-atom a-Si_3_N_4_ cell was selected. This approach disentangles the problem of site sampling from the broad range of trap sites previously described in a-Si_3_N_4_ (Fig. 1, stages 3–5 highlighted in blue).^50,51^

With the host structure defined, the statistical sampling scheme previously developed for intrinsic and H-related defects^50,51^ was extended to Li-incorporation. An exhaustive set of initial Li insertion sites was generated *via* a Voronoi tesselation centered on each atom of the host network (Fig. 1, stages 4 and 5). For each site, the Li atom was placed on the Voronoi polyhedron face with the largest area, while maintaining a minimum distance of 2.0 Å from all neighboring atoms to prevent spurious steric repulsion in the initial configuration. These 5844 distinct starting geometries were then fully relaxed at the PBE level of theory^67,68^ (Fig. 1, stage 6). During these relaxations, the Li atom is unconstrained, allowing it to settle into a local minimum within the “cage” imposed by the amorphous network; its final position is therefore not necessarily proximate to the initial reference atom. This exhaustive screening yields a comprehensive dataset of 5844 relaxed, Li-doped structures, providing a robust statistical basis for subsequent analysis. A full breakdown by Li concentration is provided in the SI (Table S2).

A Gaussian Mixture Model (GMM) was leveraged to distill the 5844-structure PBE dataset into a compact yet comprehensive subset for the HSE06 production calculations. This process ensures that the selected structures fully represent the configurational diversity of the original dataset (Fig. 1, stage 6). The GMM is particularly well-suited for this application due to its inherent flexibility in modeling the complex, multimodal distributions of local atomic environments characteristic of amorphous materials.^69–73^ For each Li concentration, the optimal number of Gaussian components, was determined by evaluating the Bayesian (BIC) and Akaike (AIC) Information Criteria, which balance model fidelity against complexity.^74–77^ From the fitted GMM, a representative ensemble was constructed by sampling configurations from the center of each Gaussian component (central modes) as well as from the tails of the distribution (high-variance outliers). This strategy ensures that the full diversity of local environments is captured. The procedure reduces the full set of 5844 PBE structures to 280 representative configurations, which then serve as the initial geometries for the HSE06 production runs. The process is then repeated across the desired compositional range Li_1_–Li_10_. The simulated concentrations (Li_*n*_, *n* = 1–10) correspond to gravimetric capacities of 4.8 mA h g^−1^ to 47.2 mA h g^−1^. The complete mapping of *n*, capacity and stoichiometry is given in Table S2 of the SI.

The statistical framework underlying this workflow is deliberately chosen to reflect the non-equilibrium nature of the initial lithiation of a-Si_3_N_4_. Li incorporation is an irreversible, path-dependent reaction that breaks ergodicity: once inserted, the network becomes confined to a hierarchy of evolving local minima and the a *priori* probability of accessing any particular minimum is unknown. Standard equilibrium (Boltzmann-weighted) statistics are therefore inapplicable.^78–82^ For every physical property we report the full distribution without applying any configurational weights. The GMM retains the character of the original sampling by weighting each identified structural motif in proportion to its population, ensuring that the final ensemble reproduces the diversity of the full energy landscape. The approach is analogous to the “inherent-structure” formalism widely used in glass physics.^83,84^

The entire workflow was implemented in Python. The ASE library^85^ was used for structure manipulation and database management, SciPy^86^ for Voronoi analysis, and scikit-learn^87^ for GMM fitting and sampling. Visualizations were generated with matplotlib,^88^ atomic structures with VMD^89^ and Voronoi polyhedra with Ovito.^90^

To characterize local distortions in the a-Si_3_N_4_ network induced by lithium incorporation, the associated strain field has been analyzed in detail. For each individual configuration containing inserted Li, the displacement vector of every Si and N atom is computed with respect to its position in Li_*n*−1_. Averaging these displacements over all configurations belonging to a given Li_*n*_ ensemble yields an average displacement vector for each atom in the structure. To visualize anisotropy in the ensemble-average strain, a continuous scalar strain density field is then constructed by superimposing asymmetric Gaussian functions centered at the undistorted atomic positions. The resulting field is defined as2
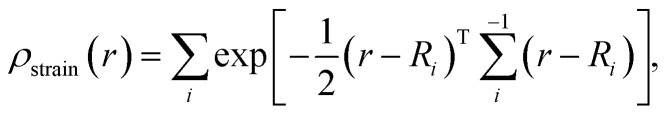
where *R*_*i*_ is the undistorted position of atom *i* and Σ_*i*_ is the covariance matrix constructed from the direction and magnitude of the associated displacement vectors. To reduce visual noise, displacements with magnitudes less than 0.1 Å are excluded from the strain density evaluation. For each Li_*n*_ ensemble the strain-density field is projected onto the crystallographic plane that shows the largest in-plane displacement, as determined from the full three-dimensional strain field. Strain contributions are averaged along its normal direction to yield a scalar field that is rendered as a heat map, with colour intensity proportional to the local strain magnitude. Projections onto other planes—whether at different *z* values or along alternative orientations—were found to add no further insight and are therefore omitted. The maps are displayed in the original simulation coordinates without re-centering relative to periodic boundary conditions.

## 3 Results and discussion

### 3.1 Initial incorporation: Li_1_

**Fig. 2:**
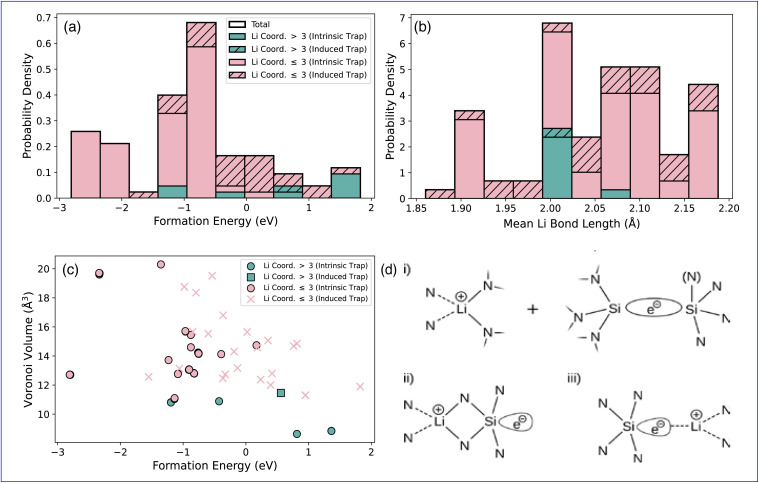
(a) Distribution of Li-formation energies in a-Si_3_N_4_. The total distribution (black outline) is subdivided based on coordination (pink and turquoise) and trapping site (crosshatched). (b) Li-bond length distribution in the first coordination shell, with the same subdivision as in (a). Histogram bars represent the probability–density estimate ; the distribution is normalised by ∫*p*(*x*) d*x* = 1. (c) Relationship between the Voronoi volumes of the incorporated Li atoms and the formation energies. (d) Schematic representation of the main Li-incorporation configurations in the a-Si_3_N_4_ network.

We first study the incorporation of a single Li in a-Si_3_N_4_. After relaxation, the incorporation environments can be classified based on the local coordination within the amorphous network and the associated electron trapping sites (Fig. 2). Li incorporation results in a wide range of structural modifications, leading to a broad distribution of formation energies. As shown in Fig. 2a, they span from −2.81 eV to 1.82 eV (referenced to lithium bulk metal–see eqn (1)). For each insertion site, the mean bond length of Li with the neighboring atoms in its first coordination shell is depicted in Fig. 2b. Typically, Li is coordinated by two or three N atoms with a bond length of approximately 2 Å. However, the incorporation of Li often results in one or more bonds being significantly extended by 10% to 20% to accommodate the Li atom. In a small fraction of cases (9.8%), steric crowding forces Li into an extended coordination environment that includes both N and Si atoms, where steric constraints prevent Li from relaxing into a more favorable configuration (Fig. 2c). As observed previously for H defects in a-Si_3_N_4_,^51^ there is a weak correlation between steric repulsion and Li formation energy, with higher formation energies associated with smaller Voronoi volumes (Fig. 2c). It is important to note that the relationship between Li geometry, formation energy, and the morphology of the a-Si_3_N_4_ network is complex and cannot be fully captured by a simple descriptor.

The incorporation of Li in a-Si_3_N_4_ is governed by the reaction at the anode: Li^0^ → Li^+^ + e^−^. The interaction of the resulting electron with the amorphous network significantly influences the mode of Li storage. This behavior is in stark contrast to that in crystalline β-Si_3_N_4_, where the electron does not localize but instead gives rise to a shallow donor state (see SI S1 and S2). This interaction allows the Li sites to be categorized into two main classes. The geometries corresponding to each trapping type are illustrated schematically in Fig. 2d, highlighting both similarities and key differences. In 73% of the sites, the electron is accommodated at an intrinsic trap site within the a-Si_3_N_4_ network, previously characterized and independent of Li except as the electron source.^50^ These sites are labeled as intrinsic traps in Fig. 2a–c and schematically depicted in Fig. 2 subpanel d : i. The remaining 27% of configurations involve electron traps that are induced by distortions in the network, driven by the presence of Li. In these cases, the electron is trapped within the coordination sphere of Li. These induced traps can be further subdivided based on whether Li induces the distortion without directly interacting with the trap site (Fig. 2dii) or directly interacts with the induced trap site (Fig. 2diii). The formation energy for Li with induced traps is significantly higher than for those with intrinsic traps, dominating configurations with energies above −0.5 eV (Fig. 2a). There is no straightforward geometric predictor to distinguish between sites that result in intrinsic *versus* induced trapping, as both types are observed across the full range of bond lengths (Fig. 2b), angles, and steric environments (Fig. 2c).

Returning to the geometries of the trapping sites, for the intrinsic traps (Fig. 2di), the electron is decoupled from the Li^+^ site. The Li^+^ is typically coordinated by 2 to 3 N atoms, forming the Li coordination shell. The Li–N bond lengths vary according to the local environment, but generally, 2 to 3 of the bonds are shorter than 2 Å, while 1 to 2 are longer, exceeding 2.5 Å. This variation results in a range of site symmetries, including C_2_, C_3_, T_d_, and their broken symmetry variants. The lowest energy configurations occur when Li^+^ interacts with 2-coordinated N atoms, due to their negative polarization relative to 3-coordinated N atoms. These low-energy sites are rare – both in experiments^34,35^ (6%) and our computational setup (1.67%).

For induced traps (Fig. 2dii and iii), the situation is more complex, as the electron-induced distortion and Li incorporation are interdependent. In Fig. 2dii, Li interacts with N atoms connected to, but not directly part of, the trap site. The presence of Li induces a distortion that creates an electron trap at a nearby Si atom. The Li coordination and Li–N separation in this case are similar to those in intrinsic traps, with 2 to 3 N atoms as nearest neighbors. However, due to the link to the trap site, a Si atom is always found as the next neighbor at a distance of approximately 2.5 Å, contrasting with the intrinsic trap scenario. Finally, in the third type of trap (Fig. 2diii), there is a direct interaction between Li and the trapping Si atom, forming a Li–Si interaction. Here, the nearest neighbor is a Si atom at a separation of 2.1 Å, with 2 to 3 N atoms at a similar distance.

**Fig. 3:**
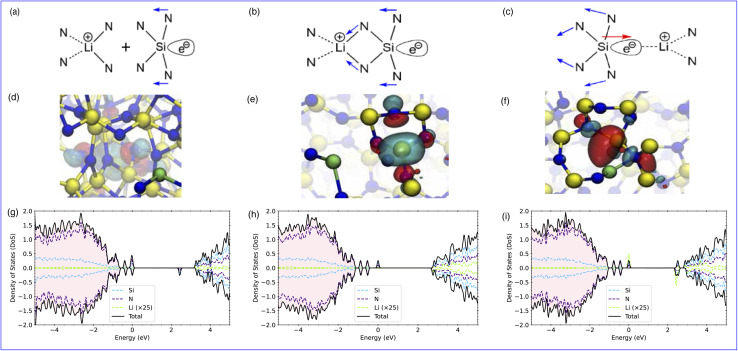
Schematics for the relaxation of each of the of electron traps showing the intrinsic trap (a), induced trap non-interacting Li (b), and the induced trap where Li is interacting (c). The corresponding occupied trap states are visualised in (d) for the intrinsic trap, (e) for the induced trap with non-interacting Li, and (f) induced trap with interacting Li. The density of states are shown in (g) for the intrinsic trap, (h) for the induced trap with non-interacting Li, and (i) induced trap with interacting Li. The filled states in (g)–(i) are indicated by the shaded region below the Fermi energy. For clarity the Li peak has been increased by a factor of 25 as indicated in the legends.

The distortion in the amorphous network is primarily driven by the electron trapping process, whether intrinsic or Li-induced. All of the traps shown in Fig. 3 share the same fundamental motif—an excess electron localised on a four-coordinated, strained Si. The distinction lies only in origin: intrinsic traps being an inherent part of the amorphous network (Fig. 3a), whereas induced traps appear only after the network is distorted by a proximate Li ion (Fig. 3b and c). The states occupied and their relationship to Li are shown in Fig. 3d–f. In the case of the intrinsic traps the Li and trap are uncorrelated (Fig. 3d), with the same trap site being occupied regardless of Li position. Whereas for the induced traps the Li is in close proximity, in the next-neighbour or next-nearest-neighbour shell of trap site (Fig. 3e and f), and a broad range of trap sites are occupied. Induced traps are further divided depending of whether the Li drives the distortion only (Fig. 3b, e and h) or whether it directly interacts with the trap site (Fig. 3c, f and i).

In both intrinsic and non-interacting induced traps, the density of states (DoS) shows an occupied state at (Fig. 3g) or near (Fig. 3h) the valence band maximum (VBM), and a related unoccupied state at (Fig. 3h) or just below (Fig. 3g) the conduction band minimum (CBM), that are predominantly Si-character trap states. The position of the states is dictated by the extent of local relaxation that can be accommodated by a given local environment. However, in the case of induced traps where Li directly interacts with the trap site, both the relaxation and DoS are distinct (Fig. 3c, f and i). This is characterized by the rearrangement of a small number of atoms representing the immediate coordination shell, with the DoS showing trap states of mixed Li and Si character. The different trapping sites exhibit markedly different Mulliken charges associated with the Li sites. As expected, Li remains cationic in all cases. In intrinsic and non-interacting induced traps, the Mulliken charges range from 0.6 e^−^ to 0.9 e^−^. However, when Li directly interacts with the trap site, there is a significant reduction in Mulliken charges, ranging from 0.2 e^−^ to 0.4 e^−^, indicating electron sharing between Si and Li and confirmed by the DoS (Fig. 3i).

### 3.2 Li_2_, Li_3_ and Li_4_ incorporation

**Fig. 4:**
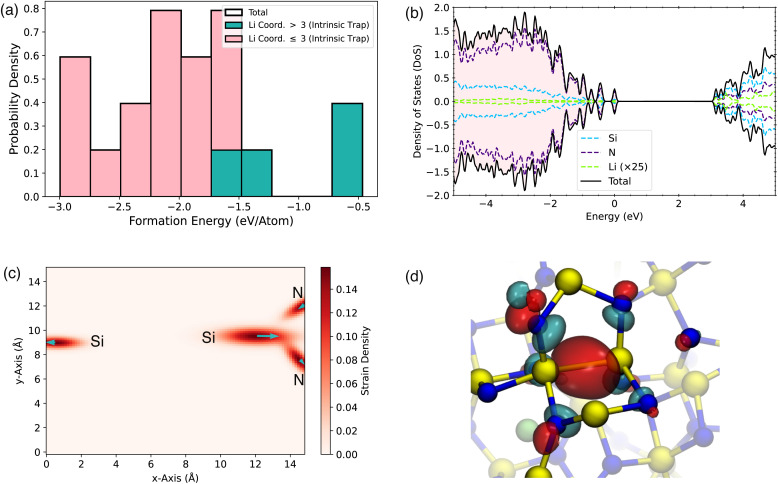
(a) Distribution of Li_2_ formation energies in a-Si_3_N_4_. As in Fig. 2a, the total distribution (black outline) is subdivided based on coordination and trapping site. Histogram bars represent the probability–density estimate 
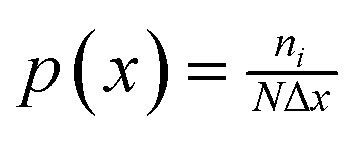
; the distribution is normalised by ∫*p*(*x*) d*x* = 1. (b) DoS showing the occupied bi-polaron state, the filled states are indicated by the shaded region below the Fermi energy. For clarity the Li peak has been increased by a factor of 25. (c) Average 2D projection of the strain induced as a result of Li_2_ incorporation and the associated bi-polaron formation. (d) Doubly occupied bi-polaron state forming a Si–Si bond with a length of 2.32 Å. Blue spheres shows nitrogen, and yellow silicon. Red iso-surface shows the spin up channel, and light blue the spin down channel.

The incorporation of a second Li atom is driven by electrostatic repulsion, maximizing the distance between the two like-charged Li^+^ ions, as might reasonably be expected. The resulting energetic landscape is strongly influenced by charge trapping: the average formation energy per Li atom becomes significantly more favourable due to bi-polaron formation (Fig. 4). In contrast to the Li_1_ case, where multiple trap sites are accessible (Fig. 2d), all Li_2_ configurations relax to a single dominant trap in which a second electron is trapped at the original intrinsic trap site (Fig. 4a). As a consequence no Li-induced traps are observed. The associated structural relaxation is pronounced: the trap-forming Si atom back-projects to form a Si–Si bond with a neighboring Si atom (Fig. 4c and d). The ensemble-averaged strain map (Fig. 4c) confirms that this distortion is consistent across configurations and decoupled from local Li-induced relaxation. Structural variations specific to individual cells are suppressed in the averaging, leaving only the common bi-polaron signature. It should be noted that the range of *E*_form_ displayed in Fig. 4a reflects the ease with which two Li ions can be incorporated into the a-Si_3_N_4_ network; with the occupied bi-polaron state constant across all configurations, the remaining variation arises solely from the local Li^+^ incorporation environment.

This reorganization modifies the electronic structure (Fig. 4b). In the single polaron case, the trapped electron localizes on a Si dangling bond, yielding occupied and unoccupied defect states in the band gap (Fig. 3d). In contrast, the bi-polaron states are approximately 0.7 eV below the valence band maximum, and no occupied states remain in the band gap. The unoccupied Li-derived states remain in the conduction band, rendering the system electronically inactive. The Si–Si bond persists after removal of the Li atoms (and/or the associated electrons), indicating that the structural rearrangement is irreversible. This mirrors the behavior previously reported for hydrogen incorporation, where a single-electron polaron relaxes irreversibly to form a bi-polaron state on the addition of a second electron.^51^

**Fig. 5:**
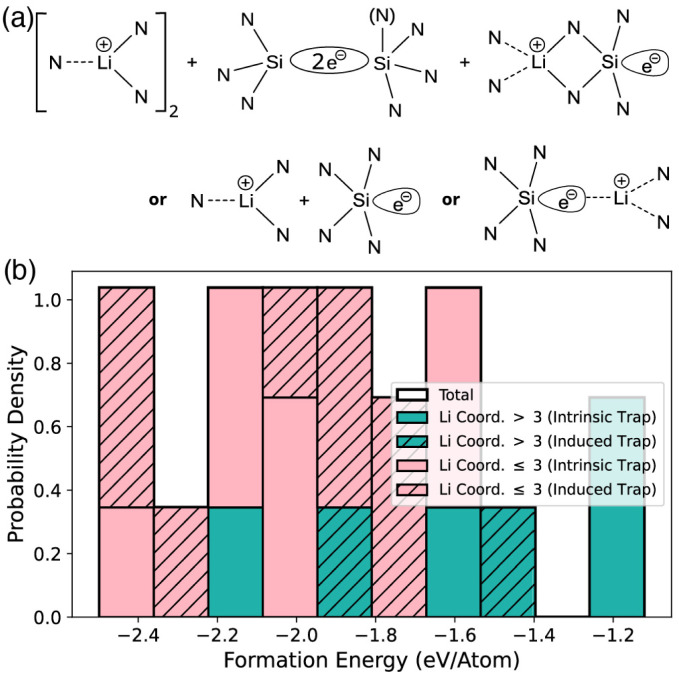
(a) Shows a schematic picture of the bi-polaron and the subsequent polaron, and (b) the distribution of Li_3_ formation energies. The total distribution (black outline) is divided by coordination (pink and turquoise) and the nature of the trapping site (crosshatched). Histogram bars represent the probability–density estimate 
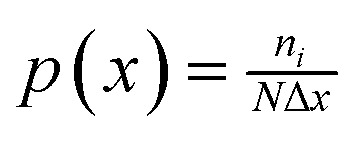
; the distribution is normalised by ∫*p*(*x*) d*x* = 1.

The Li_3_ system shares several important similarities with the Li_1_ case, with the key difference being that the lowest energy bi-polaron state in the network is occupied and therefore unavailable. This has two significant consequences. First, the distinction between intrinsic and induced traps becomes less clear-cut, as the network distortion is influenced by both the presence of Li and the bi-polaron. To maintain consistency, the following definition is applied: if trapping occurs within the extended coordination sphere (including both the N and Si coordination shells) of a Li atom, it is considered induced. Otherwise, it is classified as intrinsic. Second, the energy difference between induced and intrinsic traps becomes less pronounced. As shown in Fig. 5a and b, both types are now observed across the full range of formation energies rather than being confined to higher energy configurations as seen in Fig. 2a. The lower energy configurations continue to favor Li coordination by 3 or 2 N atoms, with an increased occurrence of Li sites exhibiting coordination numbers greater than 3. The intrinsic and induced traps can still be divided into the broad categories described for Li_1_. The main difference is that instead of a single intrinsic trap site, there are now three approximately iso-energetic sites. It is important to note that the formation energy results from both electron trapping and the accommodation of Li^+^ within the lattice.

In each case, trapping occurs at an existing wide bond angle within the amorphous network, where the electron localizes. The induced traps exhibit the same behaviors, with Li either inducing a distortion that leads to electron trapping within the Li coordination sphere or directly interacting with the trap site. Both of these configurations have already been illustrated in Fig. 5a and are the same as those shown in Fig. 2d. The presence of the existing electron trap in the amorphous network does not impact Li storage or electron trapping beyond the geometric distortions it induces. Electronically, the Si–Si bi-polaron states remain within the valence band (Fig. 4b) and are thus electrically inactive with respect to both Li^+^ and charge redistribution within the network.

**Fig. 6:**
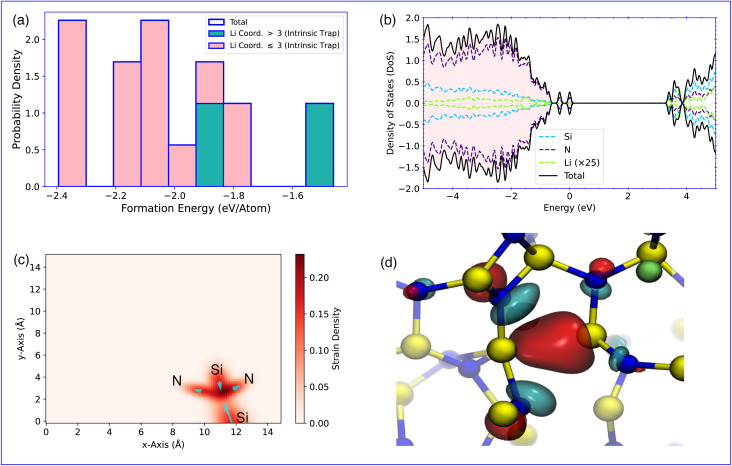
(a) Distribution of Li_4_ formation energies in a-Si_3_N_4_, the total distribution (black outline) is divided by coordination and trapping site. Histogram bars represent the probability-density estimate 
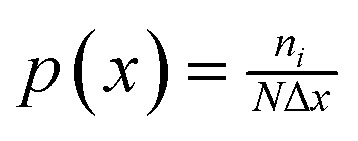
; the distribution is normalised by ∫*p*(*x*) d*x* = 1. (b) DoS showing the occupied bi-polaron state, the filled states are indicated by the shaded region below the Fermi energy. For clarity the Li peak has been increased by ×25. (c) A 2D projection of the strain induced as a result of Li_4_ incorporation and the associated bi-polaron formation. (d) The doubly occupied bi-polaron state forming a Si–Si bond with a length of 2.4 Å. Blue spheres shows nitrogen, green lithium, and yellow silicon. Red iso-surface shows the spin up channel, and light blue the spin down channel.

The Li_4_ system exhibits several important similarities with Li_2_, with the lowest energy bi-polaron already filled, and the next lowest energy configuration also occupied. As before, a single bi-polaron forms independently of the Li geometry and its position relative to the trap site. However, unlike in the Li_2_ case, there is no significant decrease in *E*_form_; rather, the energy distribution of Li_4_ configurations is tighter, with a greater number of low-energy states (Fig. 6a). The second bi-polaron shows similar relaxation behavior, although it is constrained by a different local environment. Specifically, one Si center back-projects to form an Si–Si interaction, accompanied by an outward relaxation of the neighboring N atoms to accommodate this change. This relaxation is depicted in Fig. 6c, which shows the Si centers moving towards each other and the displacement of the adjacent N atoms. The DoS for Li_4_ (Fig. 6b) also reveals a defect free band gap, similar to Li_2_, with the bi-polaron states situated below the valence band maximum (VBM). The doubly occupied trap state, shown in Fig. 6d, features the same Si–Si bonding interaction observed in the Li_2_ system.

### 3.3 Increasing Li concentration

**Fig. 7:**
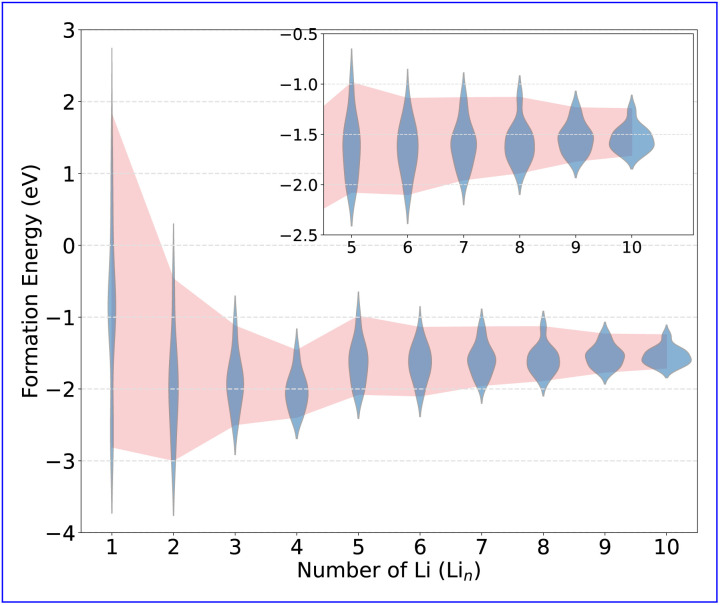
Progression in formation energy as a function of number of Li in the range Li_1_ to L_10_. For each concentration, the violin plot shows the underlying distribution resulting from the structural ensemble. The red envelope function provides a guide for the eye. The inset zooms into the concentration range from Li_5_ to Li_10_.

As the Li concentration increases, the pattern observed in Li_1_ through Li_4_ is continued, with a diverse range of single-electron traps giving way to bi-polaron states. The consequences for the formation energies are shown in Fig. 7: initially, there is a strong energetic driver for bi-polaron formation, with a marked drop in formation energy from Li_1_ to Li_2_, driven by the collapse of the broad variety of electron traps described in Section 3.1 into a single bi-polaron configuration. A similar trend is seen from Li_3_ to Li_4_, favoring bi-polaron formation, although the magnitude of this stabilization is greatly reduced.

Li_4_ represents the filling of the last intrinsic bi-polaron site in the host lattice. For Li_5_ and above, the trap states are a consequence of Li-incorporation and the resulting structural modifications (resulting from the Li and electron trapping). This shift from filling intrinsic to creating induced trap states is accompanied by an increase in energy from Li_4_ to Li_5_. The trend then echoes the behavior of the intrinsic trap states (Li_1_ to Li_4_): a wide distribution of polaron states collapses into a single bi-polaron configuration (at Li_6_). In each case the bi-polaron states impart an irreversible change on the lattice, creating Si–Si bonded motifs.

Beyond Li_7_, a single dominant bi-polaron configuration no longer exists; instead, there are three to four geometrically distinct configurations. These all exhibit similar relaxation behavior, forming an Si–Si bond with lengths in the range of 2.2 Å to 2.4 Å, and represent the onset of more complex, polyfurcation of bi-polaron formation. Looking at the electronic structure, while all bi-polaron states reside below the VBM, there is a clear shift up towards the VBM as the Li concentration increases.

## 4 Discussion and summary

The initial incorporation of Li into a-Si_3_N_4_ induces significant structural modifications through the formation of various one- and two-electron trap states, as governed by the reaction:

3 *n*Li_0_ → *n*Li^+^ + *n*e^−^_trap_

**Fig. 8:**
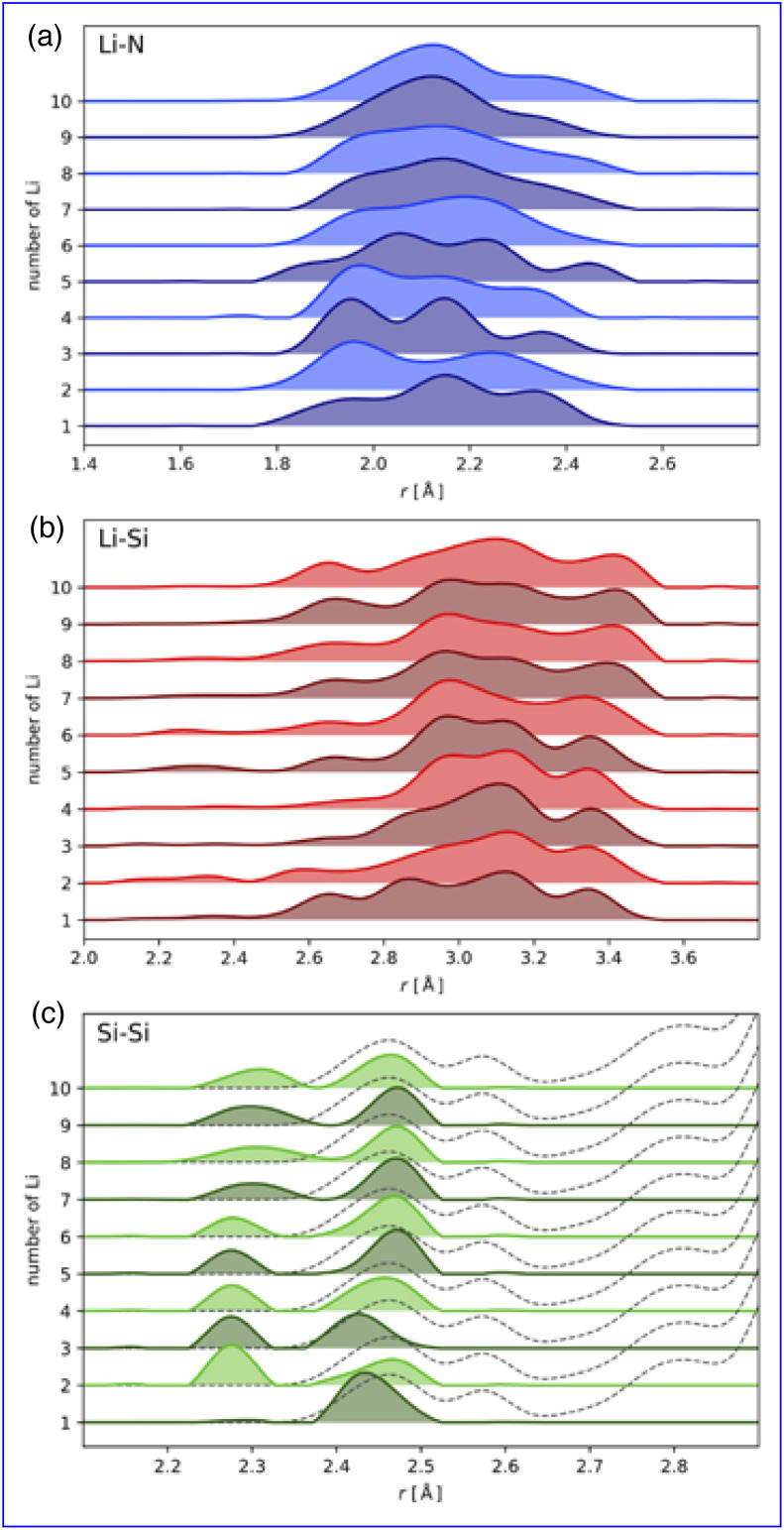
Bond length distributions as a function of Li concentration for (a) Li–N and (b) Li–Si. (c) Distributions for the short Si–Si pair distances that result from bi-polaron formation and the precursor polaron states. The dashed line reflects the full Si–Si pair distribution as shown in the SI S3b. All histograms are empirical, equal-weight distributions of the sampled configurations (no re-weighting by energy).

Despite the wide range of local environments within the amorphous network, common structural features emerge, as shown in Fig. 8. A broad distribution of Li–N bonds are found ranging from 1.8 Å to 2.5 Å (Fig. 8a), while Li–Si bond lengths, which are spread over a wider range of 2.0 Å to 3.6 Å (Fig. 8b), are more sensitive to the Li-concentration (Fig. 8b). The secondary Li–Si feature arises from Si atoms in the second coordination shell, with bond lengths above 3.0 Å, seen in both induced and intrinsic traps. Finally, the Si–Si bond lengths show a broad distribution centered at 2.45 Å at Li_1_ (Fig. 8c), representing the previously described polaron states. These are significantly shorter than the Si–Si first-shell maximum at 3.5 Å (see SI Fig. S3b). At Li_2_ and above, the distribution becomes bi-modal, with the bi-polaron states at ≈2.25 Å and a distribution of precursor states below 2.5 Å (Fig. 8c). As Li concentration increases, these features evolve, as shown in Fig. 8. Notably, the Li–N peak broadens significantly after Li_4_, coinciding with an upward shift of the formation-energy distribution (Fig. 7), indicating a transition from intrinsic to induced trapping, where Li-induced network distortions facilitate electron trapping. Concurrently, the Li–Si bond length range broadens as induced traps become more prevalent, driving the skew to shorter Li–Si separations at higher Li concentrations. The bi-polaron becomes more pronounced as more Li atoms are incorporated, transitioning from single polaron to bi-polaron states.

The progression from intrinsic to induced trapping follows a clear sequence. Initially, those intrinsic traps, which are naturally present in the network, get filled, followed by the formation and occupation of induced traps. The trapping of 2e^−^ drives an irreversible network relaxation, forming Si–Si bonds, which in turn drives the sequential occupation of all intrinsic traps in the system. In the reference cell two intrinsic bi-polarons are able to relax. Once these intrinsic traps are saturated, the induced traps are filled – both polarons and bi-polarons. Although induced traps have higher formation energies than intrinsic traps, they remain significantly favored with respect to the delocalised state, especially at higher Li concentrations. This irreversible structural modification—marked by the creation of Si–Si formation—mirrors earlier findings in a-Si_3_N_4_ under hydrogen incorporation.^51^ This stepwise process provides the atomistic basis for the formation and stability of the Si-rich regions during the initial lithiation. The observed relationship between stoichiometry and Li storage can be understood through these trap states. It is well-established that Si-rich a-Si_3_N_4_ increases the concentration of electron trap states,^91–93^ a trend further enhanced by the presence of Si…Si precursor states capable of trapping electrons.^28,94^ This characteristic likely contributes to the capacity fade observed as sub-stoichiometry increases, where the network's ability to accommodate distortions diminishes. However, the models used in this study are all stoichiometric. Therefore, caution should be exercised when extrapolating these findings to significantly sub-stoichiometric compositions unless similar structural features are present.

**Fig. 9:**
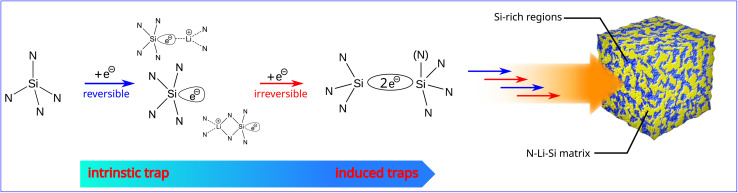
Schematic representation of the initial Li-incorporation, progressing *via* a number of reversible (polaron) and irreversible (bi-polaron) states.

The structural ensembles generated in this study align well with previously reported experimental data.^43,47,49^ Through deconvolution of the PDF, with residuals from reported fits for both lithiated and delithiated samples, showing good agreement with the Li structural features given by the superimposition of Fig. 8a and b. These features include a broad peak between 1.9 Å to 2.2 Å, a sharp peak with negative skew at 2.5 Å, a secondary peak at 2.8 Å, and a broad peak centered around 3.0 Å. The presence of these features in both lithiated and delithiated (along with their absence in the pristine) samples suggests a multi-stage matrix-forming process. Initially, Li incorporation populates intrinsic electron trap states in the network; once these states are filled, a shift to induced trapping occurs. In both cases, the formation of bi-polaron states results in Si–Si rich regions, leading to irreversible changes in the amorphous network. This behavior is in agreement with the experimental EDX which shows a clustering and redistribution of Si post lithiation.^47^ As additional Li is incorporated, the final matrix forms *via* a number of intermediate compositions, as illustrated schematically in Fig. 9.

The electronic structure of a-Si_3_N_4_ during Li incorporation is characterized by the formation of distinct polaron and bi-polaron states, which play an important role in determining the material's electronic and electrochemical properties. Initially, single polaron states dominate, with occupied states close to the VBM (0 eV to 0.8 eV), and empty states close to the CBM (0 eV to −0.7 eV). These give way to form bi-polaron configurations with occupied states in the valence band. As the Li-concentration further increases, these bi-polaron states increase in energy, becoming near-degenerate with the VBM. This evolution progresses from isolated traps to an interconnected polaron networks underscoring the complex interplay between Li incorporation, local environment, network relaxation, and the resulting electronic properties. It is this coupling that resolves the apparent contradiction of a matrix that is simultaneously redox-active and inert: polaron states enable reversible, electrochemically active behavior, while bi-polaron states drive irreversible structural reorganization and are electronically inactive (Fig. 9).

Finally, the parallels between a-Si_3_N_4_, a-SiO_2_ and other sub-stoichiometric oxides are noteworthy. Both exhibit intrinsic charge trapping, leading to electron polaron and bi-polaron formation,^95,96^ and have similar Li incorporation geometries. This suggests a potentially general mechanism for conversion-type anodes based on oxides and nitrides, where intrinsic charge trapping drives bi-polaron formation, causing irreversible modifications to the amorphous network and initiating the matrix formation process. Extending these findings beyond a-Si_3_N_4_ is currently under investigation, however, the observed parallels provide a strong foundation for further study.

## 5 Conclusions

This study provides important insights into the structural and electronic behavior of a-Si_3_N_4_ during the initial Li incorporation, highlighting the formation and evolution of polaron and bi-polaron states. These states play a pivotal role in determining the material's electrochemical properties, particularly in the context of lithium-ion battery anodes. The transition from single polaron to bi-polaron states and the associated changes in structure are vital in generating the initial Si-rich regions of the network. Our findings suggest that managing the distribution and energy of these trap states could be key to optimizing a-Si_3_N_4_-based anodes for improved capacity and cycling stability.

## Conflicts of interest

There are no conflicts to declare.

## Supplementary Material

TA-013-D5TA04013B-s001

## Data Availability

All structures generated in this study are available *via*https://www.zenodo.orghttps://doi.org/10.5281/zenodo.15297234, together with input parameters and specifications for the compilation of the CP2K package. The supporting information contains Li-incorporation in β-Si_3_N_4_, properties of the pristine cell, and characterisation of Li incorporation sites. See DOI: https://doi.org/10.1039/d5ta04013b.
