# Supplementary material for: Forged by charge: polaron-induced matrix formation in silicon nitride conversion-type anodes for lithium-ion batteries
Source: J Mater Chem A Mater. 2025 Aug 4;13(40):34260–72. doi: 10.1039/d5ta04013b (PMC12379599; doi:10.1039/d5ta04013b)
Supplement: TA-013-D5TA04013B-s001 [file TA-013-D5TA04013B-s001.pdf]

Supplementary Information (SI) for Journal of Materials Chemistry A.
This journal is © The Royal Society of Chemistry 2025

Supporting Information: Forged by Charge: Polaron-Induced Matrix Formation in Silicon Nitride Conversion-Type Anodes for Lithium-ion Batteries

Jonathon Cottom,[†,‡,¶] Lukas Hückmann,[¶] Jörg Meyer,[¶] and Emilia Olsson[*,†,‡]

†*Advanced Research Center for Nanolithography, Science Park 106, 1098 XG Amsterdam, The Netherlands*

‡*Institute for Theoretical Physics, University of Amsterdam, Science Park 904, Amsterdam, 1098 XH, the Netherlands*

¶*Leiden Institute of Chemistry, Gorlaeus Laboratories, Leiden University, P.O. Box 9502, 2300 RA Leiden, The Netherlands*

E-mail: k.i.e.olsson@uva.nl

Contents

1 Lithium Incorporation in β-Si_3N_4 **S-3**

2 Properties of the Pristine Cell **S-5**

2.1 Ensemble Properties S-5

2.2 Polaron and Bi-Polaron Charge Trapping S-7

3 Statistical Sampling of Lithium Incorporation Sites **S-8**

References **S-11**

1 Lithium Incorporation in β-Si_3N_4

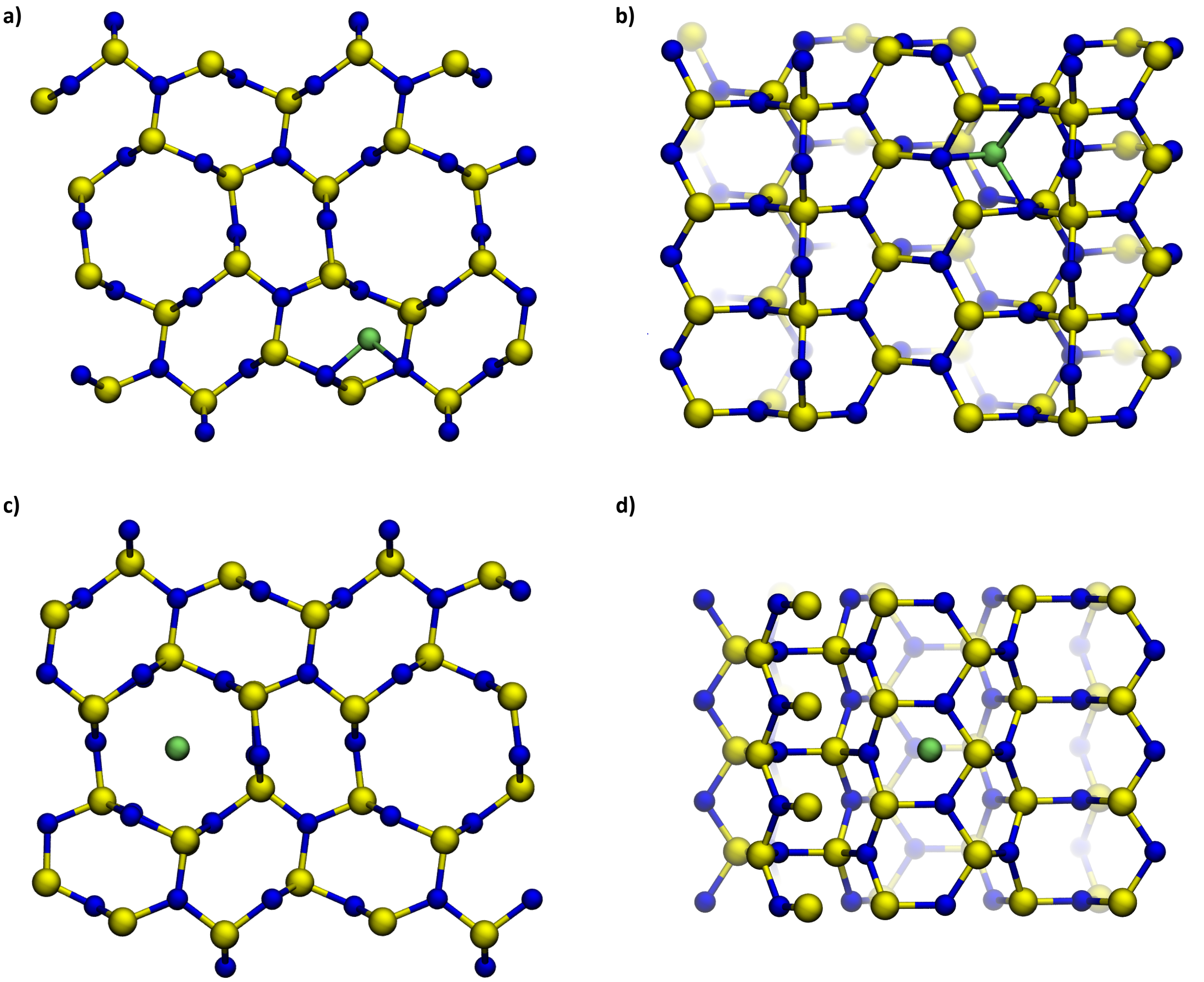

Figure S1: Depiction of the two Li-incorporation sites in β-Si_3N_4 with a)/b) Li being in the narrow site and c)/d) Li being in the wide site. The viewing direction of a)/c) is along the z-axis and b)/d) provide a side view of the simulation cell. All images show electrically neutral cells. Silicon is colored yellow, nitrogen is blue, and lithium is green.

The structure of β-Si_3N_4 comprises layers of eight- and twelve-membered rings in the ab-plane tightly linked in c-direction. The rings are stacked such that they form wide and narrow tubes in c-direction leading to two inequivalent Li incorporation sites, see Figure S1.

In the neutral charge state (Li^0, $q = 0$), lithium acts as a shallow donor, donating an electron to the conduction band while remaining in the middle of the cavity in both incorporation sites. The Li cation remains weakly bound in the cavity, with minimal displacement

of neighboring Si or N atoms.

$$\mathrm{Li}^0 + \mathrm{Si_3N_4} \longrightarrow \mathrm{Si_3N_4}^- + \mathrm{Li}^+ \ . \tag{1}$$

The electrostatic interaction with negatively polarized nitrogen atoms causes Li in the narrow site to shift slightly towards the surrounding anions, reducing the Li–N distances to the range of 1.81 Å to 1.98 Å. However, the formation energy (E_{form}) is significantly higher for Li in the narrow site (4.56 eV) than in the wide site (2.37 eV), suggesting that steric hindrance plays a significant role in destabilizing the confined interstitial site.

The charged defects are treated with the Lany-Zunger correction scheme[S1] as outlined in[S2,S3] For the majority of the bandgap, the +1 charge state is favored, as indicated by the formation energy behavior in Figure S2 for both Li-configurations. At the wide site, the neutral charge state only becomes favored for Fermi level positions near degenerate (CBM - 0.04 eV) with the CBM. By contrast, the confinement of the narrow-site Li induces negative-U behavior, with the (+1/-1) charge transition level at 5.17 eV.

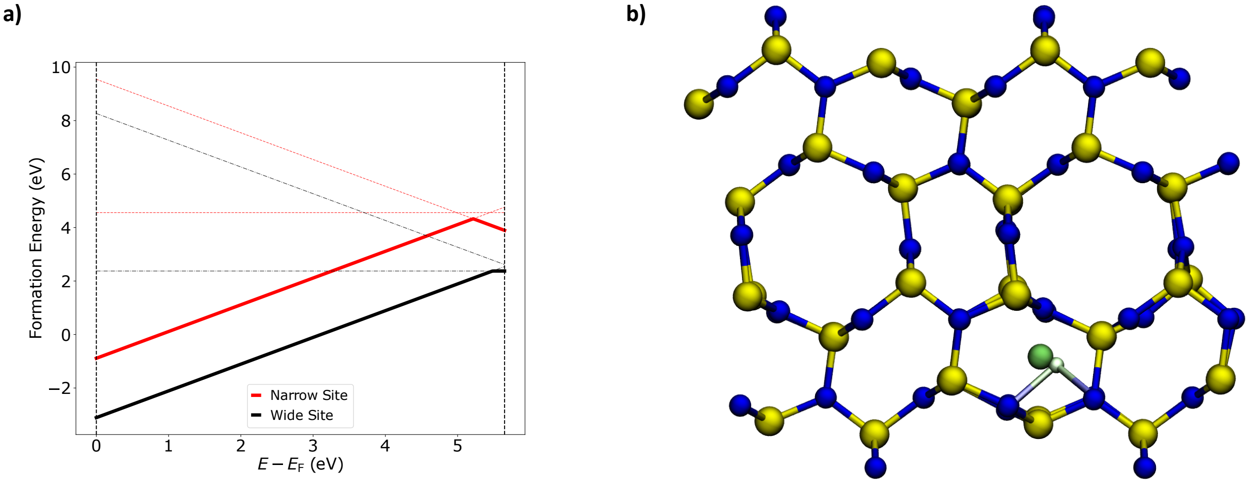

Figure S2: a) Formation energies E_{form} as a function of the Fermi level E_{F} for the Li defect sites in the narrow (red) and in the wide sites (black) in the +1, neutral, and −1 charge states. b) The narrow site in the $q = -1$ configuration where Li relaxes towards the middle of the tube. For comparison, the neutral Li is depicted in faded green.

2 Properties of the Pristine Cell

2.1 Ensemble Properties

For this study, a single a-Si_3N_4 cell from the library generated in our previous work[S2] is selected to ensure a single reference for all Li insertions. For completeness, the properties of the pristine, lithium-free cell are listed in Section 2.1 together with the average values of the entire ensemble and – if available – literature data.

Table S1: Properties of the pristine a-Si_3N_4 cell used in this study compared to ensemble averages from our previous study.[S2] Values for the density ρ, the coordination numbers CN, the band gap E_{bg}, and the trapping energies of holes E_{h^+} and electrons E_{e^-}. are obtained at the HSE06[S4,S5] level, whereas the bulk modulus B is from calculations with the MG2[S6] force field. Literature values are provided in addition if available.

Property	Unit	This study	Ensemble	Literature	
ρ	$\mathrm{g\,cm^{-3}}$	2.9	2.9	2.6 to 3.0	[S7]
CN(Si)		4.00	3.96	3.70	[S8]
CN(N)		2.98[a]	2.87	2.78	[S8]
B	GPa	159	166	156 to 161	[S9]
E_{bg}	eV	4.10	4.43	4.77	[S10]
E_{h^+}	eV	−0.58	−0.35	−0.9 to −1.4	[S11]
E_{e^-}	eV	−0.53	−0.74	−1.2 to −1.7	[S11]
ϵ		8.45		7.0 to 10.5	[S12]
c_{sh}		-0.369	–	–	[S1]
L	Å	14.72	–	–	
Trap Type[b]		intr.	–	–	

[a] $\hat{=}$ four 2-coordinated N atoms; 160 N atoms in total.
[b] see Section 2.2.

In the context of Li incorporation, the geometry of the SiN_4-tetrahedra plays a decisive role as they are subject to deformation due to the strained, disordered amorphous network they are embedded and thus altering the physical behavior of the amorphous phase compared to the crystal. The deformation of the tetrahedra is quantified by determining the opening angle of the tetrahedra faces via the cone-angle method introduced in Hückmann *et al.*[S2]. As depicted in Figure S3a, the majority of tetrahedra exhibit at least one face that is opened with respect to the ideal tetrahedron (ϕ_0 = 70.53°), facilitating Li to either coordinate

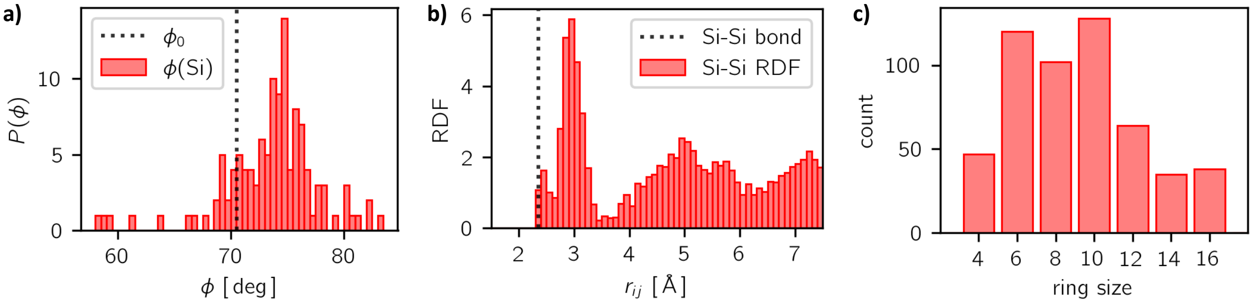

Figure S3: Selected observables of the pristine a-Si_3N_4 cell: a) The cone angle distribution $P(\phi)$ of the Si atoms, b) the Si-Si radial distribution function (red bars) with the equilibrium Si-Si bond length (dotted black), and c) the ring size distribution.

the spatially more densely packed N atoms at the backside of the particular tetrahedron or to interact with the Si atom directly. Moreover, the Si-Si radial distribution function (RDF) shows the occurrence of near-distant Si-Si pairs of ≈2.5 Å before the main peak at 3 Å (Figure S3b). These features are associated with four-membered rings found in the cell (Figure S3c), which have a diameter of approximately 2.5 Å this accords well with prior work.[S13]

2.2 Polaron and Bi-Polaron Charge Trapping

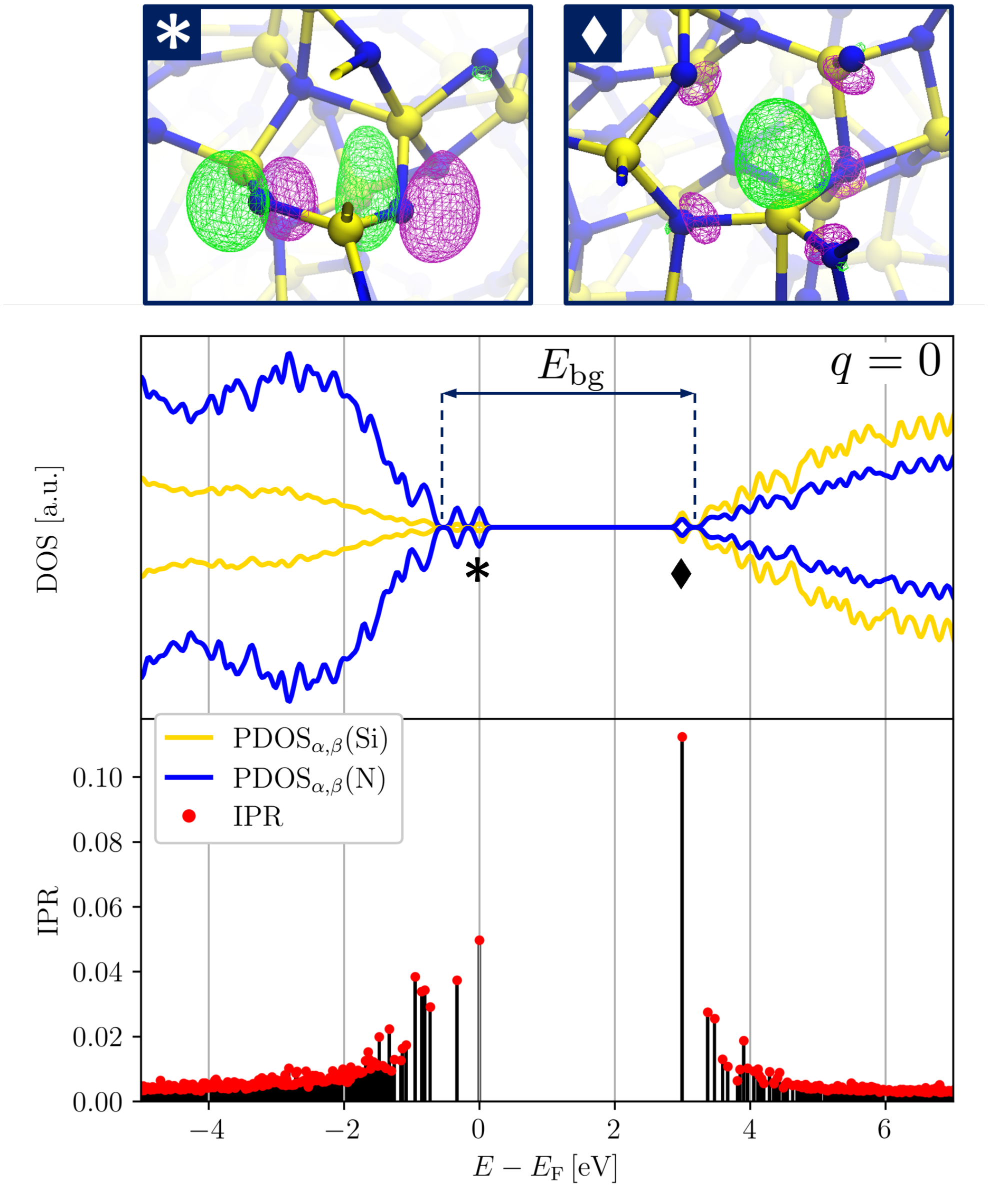

Figure S4: Upper panel: Illustration of the band edge states localized on two-coordinated nitrogen atoms above the VBM ($*$) and localized on distorted Si-tetrahedron below the CBM (♦). The isosurfaces of the spin density are set to $0.05\,e\,\mathrm{Å}^{-3}$. Middle panel: The projected electronic density of states (DOS) of Si (yellow) and N (blue) in a single neutral a-Si_3N_4 configuration. The band gap E_{bg} is measured by excluding the localized states at the band edges, with $*$, ♦ marking the intrinsic hole and electron traps respectively visualised in the top panel. Lower panel: The corresponding inverse participation ratio for all eigenstates with $-5\,\mathrm{eV} < E - E_{\mathrm{F}} < 7\,\mathrm{eV}$. For more details, see Hückmann *et al.*[S2].

3 Statistical Sampling of Lithium Incorporation Sites

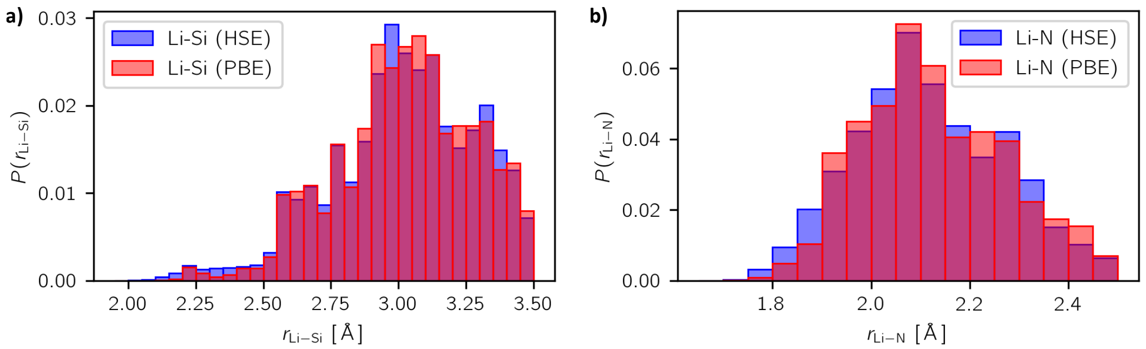

Figure S5: Screening of the statistical completeness of the Si_3N_4:Li ensemble: The distributions P of a) the Li–Si and b) the Li–N bond lengths of the PBE (red) and HSE ensemble (blue).

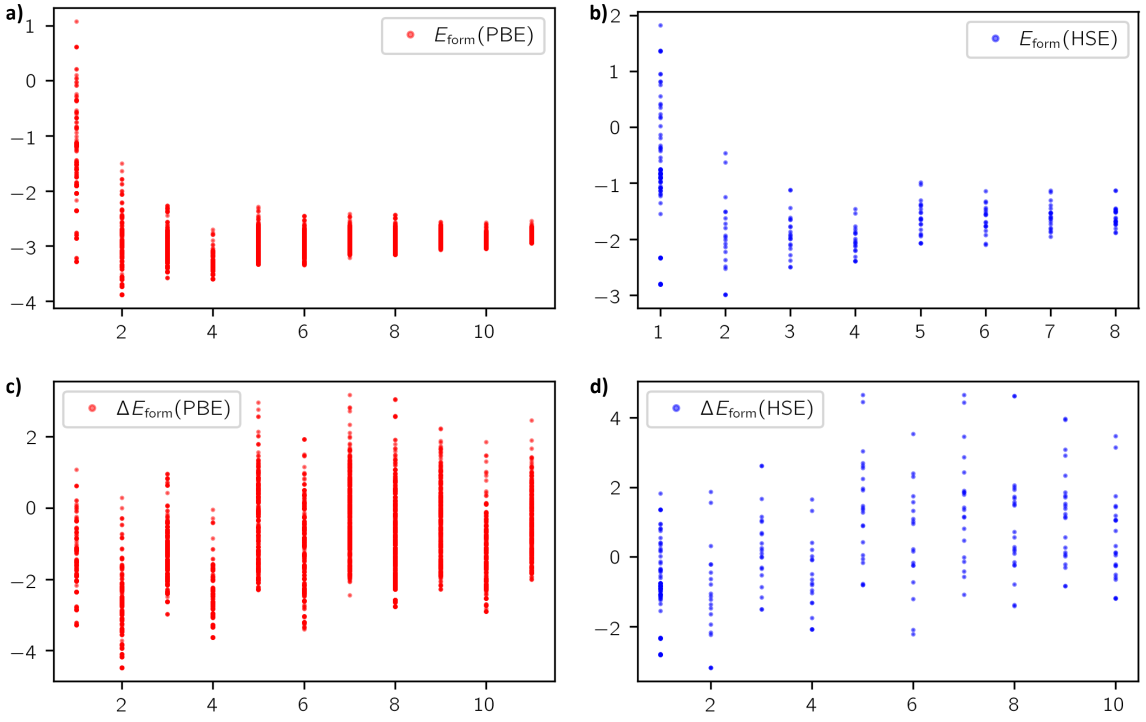

Figure S6: a)/b) The formation energy E_{form} and c)/d) the stepwise formation energy ΔE_{form} as a function of the number Li atoms (N_{Li}) on PBE (red) and HSE level (blue).

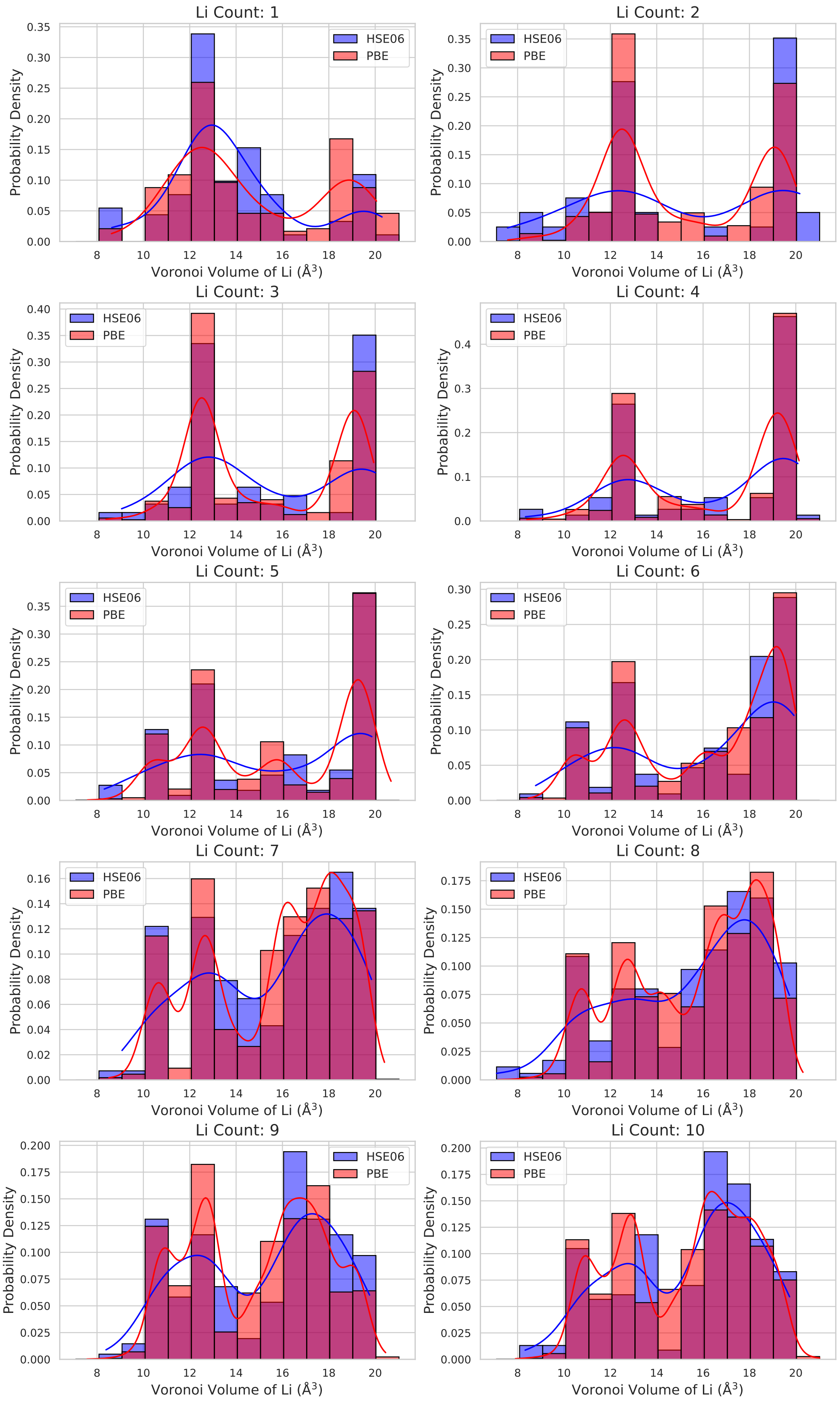

Figure S7: The volume distribution of the Voronoi polyhedra of the Li atoms of the PBE (red) and HSE (blue) optimized cells.

Table S2: Table detailing the stoichiometry, capacity and ensemble sizes considered in this work

**Number of Li**	**Formula**	**Capacity (mAh/g)**	**PBE Structures**	**HSE06 Structures**
1	$Li_{0.025}Si_3N_4$	4.8	240	92
2	$Li_{0.05}Si_3N_4$	9.5	476	20
3	$Li_{0.075}Si_3N_4$	14.3	716	21
4	$Li_{0.1}Si_3N_4$	19.0	241	19
5	$Li_{0.125}Si_3N_4$	23.8	488	22
6	$Li_{0.15}Si_3N_4$	28.5	488	18
7	$Li_{0.175}Si_3N_4$	33.2	724	20
8	$Li_{0.2}Si_3N_4$	37.8	978	22
9	$Li_{0.225}Si_3N_4$	42.5	493	23
10	$Li_{0.25}Si_3N_4$	47.1	496	23

References

(S1) Lany, S.; Zunger, A. Accurate prediction of defect properties in density functional supercell calculations. *Model. Simul. Mat. Sci. Eng.* **2009**, *17*, 084002, DOI: 10.1088/0965-0393/17/8/084002.

(S2) Hückmann, L.; Cottom, J.; Meyer, J. Intrinsic charge trapping and reversible charge induced structural modifications in a-Si_3N_4. *Adv. Phys. Res.* **2023**, 2300109, DOI: 10.1002/apxr.202300109.

(S3) Cottom, J.; Hückmann, L.; Olsson, E.; Meyer, J. From Jekyll to Hyde and Beyond: Hydrogen's Multifaceted Role in Passivation, H-Induced Breakdown, and Charging of Amorphous Silicon Nitride. *J. Phys. Chem. Lett.* **2024**, *15*, 840–848, DOI: 10.1021/acs.jpclett.3c03376.

(S4) Heyd, J.; Scuseria, G. E.; Ernzerhof, M. Hybrid functionals based on a screened Coulomb potential. *J. Chem. Phys.* **2003**, *118*, 8207–8215, DOI: 10.1063/1.1564060.

(S5) Heyd, J.; Scuseria, G. E.; Ernzerhof, M. Erratum: "Hybrid functionals based on a screened Coulomb potential" [J. Chem. Phys. 118, 8207 (2003)]. *J. Chem. Phys.* **2006**, *124*, 219906, DOI: 10.1063/1.2204597.

(S6) Marian, C. M.; Gastreich, M.; Gale, J. D. Empirical two-body potential for solid silicon nitride, boron nitride, and borosilazane modifications. *Phys. Rev. B* **2000**, *62*, 3117–3124, DOI: 10.1103/PhysRevB.62.3117.

(S7) Chu, T. L.; Lee, C. H.; Gruber, G. A. The preparation and properties of amorphous silicon nitride films. *J. Electrochem. Soc.* **1967**, *114*, 717–722, DOI: 10.1149/1.2426715.

(S8) Misawa, M.; Fukunaga, T.; Niihara, K.; Hirai, T.; Suzuki, K. Structure characterization of CVD amorphous Si_3N_4 by pulsed neutron total scattering. *J. Non-Cryst. Solids* **1979**, *34*, 313–321, DOI: 10.1016/0022-3093(79)90018-8.

(S9) Khan, A.; Philip, J.; Hess, P. Young's modulus of silicon nitride used in scanning force microscope cantilevers. *J. Appl. Phys.* **2004**, *95*, 1667–1672, DOI: 10.1063/1.1638886.

(S10) Resende, J.; Fuard, D.; Le Cunff, D.; Tortai, J.-H.; Pelissier, B. Hybridization of ellipsometry and energy loss spectra from XPS for bandgap and optical constants determination in SiON thin films. *Mater. Chem. Phys.* **2021**, *259*, 124000, DOI: 10.1016/j.matchemphys.2020.124000.

(S11) Gritsenko, V. A.; Perevalov, T. V.; Orlov, O. M.; Krasnikov, G. Y. Nature of traps responsible for the memory effect in silicon nitride. *Appl. Phys. Lett.* **2016**, *109*, DOI: 10.1063/1.4959830.

(S12) Kang, G.; Lee, D.; Lee, K.; Kim, J.; Han, S. First-principles study on the negative-U behavior of K centers in amorphous Si_3N_4-x. *Phys. Rev. Appl.* **2018**, *10*, 064052, DOI: 10.1103/PhysRevApplied.10.064052.

(S13) Vedula, R. P.; Anderson, N. L.; Strachan, A. Effect of topological disorder on structural, mechanical, and electronic properties of amorphous silicon nitride: An atomistic study. *Phys. Rev. B* **2012**, *85*, 205209, DOI: 10.1103/PhysRevB.85.205209.
